# Effect of diamine oxidase (DAO) enzyme dietary supplementation in subjects with insomnia symptoms and single nucleotide polymorphisms of the *AOC1* gene: a randomized double-blind placebo-controlled study

**DOI:** 10.1016/j.sleepx.2025.100167

**Published:** 2025-12-01

**Authors:** Júlia Ferrer-Garcia, Maria D. Navarro, Montserrat Abadias, Raquel López-García, Anna Sansalvador, Georgia Gris, Karol Uscamaita

**Affiliations:** a AdSalutem Institute for Healthy Sleep, 08008, Barcelona, Spain; b Patients Participation and Training Area, Sant Joan de Déu Barcelona Hospital, 08950, Esplugues de Llobregat, Spain; c Neurology Service, Sleep Disorders Unit, Hospital Universitari Sagrat Cor, Grupo Quirónsalud, 08034, Barcelona, Spain; d Medicine Department, University of Barcelona, 08036, Barcelona, Spain

**Keywords:** Diamine oxidase, Insomnia, Single nucleotide polymorphism, *AOC1* gene, Melatonin

## Abstract

**Objective:**

This exploratory study was designed to assess the effect of a diamine oxidase (DAO) enzyme supplement on insomnia symptoms in patients with alterations of the *AOC1* gene, which encodes the DAO enzyme.

**Methods:**

Prospective randomized, double-blind placebo-controlled study. Patients were randomized (1:1:1:1) to 28-day supplementation with the DAO product (12.6 mg/day) or placebo. The Insomnia Severity Index (ISI) and the Pittsburg Sleep Quality Index (PSQI) were completed.

**Results:**

We studied 101 patients (DAO group, n = 50; placebo, n = 51) (73.4 % women, mean age 48.3 years). Decreases in ISI scores were similar in both groups, but severe insomnia at day 28 was higher in the placebo group (5.9 % vs. 2 %). Improvement of PSQI at day 28 was higher in the DAO group (mean [SD] change −1.62 [3.45] vs. −1.47 [3.21]). Improvements of at least 1 point of PSQI in various subscales were higher in the DAO group. Also, in the DAO group and the once-daily regimen, sleep efficiency and use of sleep medication showed significant improvements vs. baseline (mean change of −0.71 [1.43], *p* = 0.023 and −0.54 [1.14], *p* = 0.043, respectively). In melatonin users, improvements in ISI at day 7 were higher in the DAO group and persisted until day 28. Overall PSQI and sleep efficiency improved significantly in the DAO group only.

**Conclusions:**

In this exploratory study, the use of a DAO supplement for 28 days improved insomnia symptoms in the presence of genetic variants of the *AOC1* gene and showed a synergy with melatonin.

Registered in the ClinicalTrials.gov (NCT07027943).

## Highlights

• This study evaluated a diamine oxidase (DAO) supplement vs. placebo for insomnia.
• The study patients had alterations of the *AOC1* gene, which encodes the DAO enzyme.
• DAO supplementation improved severe insomnia symptoms especially sleep efficiency.
• The DAO supplement showed a potential synergy with melatonin.
• DAO supplementation for 28 days was superior to placebo for improving insomnia.

## Graphical abstract

**Figure:**
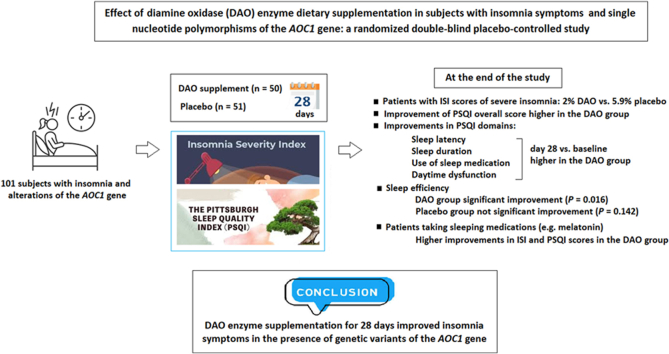

## 1 Introduction

Chronic insomnia is a prevalent sleep disorder characterized by a difficulty falling asleep, maintaining sleep consistency or experiencing early awakening, which must be present for at least 3 days a week for 3 months to meet the diagnostic criteria of the American Academy of Sleep Medicine's International Classification of Sleep Disorders, Third Edition (ICSD-3-TR) [1]. Importantly, insomnia occurs despite adequate opportunities to sleep and is accompanied by daytime symptoms. Chronic insomnia is frequently underdiagnosed and undertreated and remains an important public health problem worldwide [2,3]. The prevalence estimates of chronic insomnia vary widely. In industrialized countries, about 6 % of adults suffer from chronic insomnia [4]. However, when considering the prevalence of people who report suffering from one or more of the insomnia symptoms, i.e., difficulty initiating sleep, difficulty maintaining sleep, and early waking up with inability to continue sleeping, the estimates increase up to 30 % [5]. In a representative sample of the Spanish population of 4065 individuals aged 15 years or older, insomnia affected up to one in five individuals, with a prevalence of 20.8 % [6].

Chronic insomnia can be a clinical manifestation of various diseases, including impaired degradation of exogenous histamine. Moreover, histamine, acting via H_1_ and/or H_3_ receptor has a pivotal role in sleep-wakefulness regulation [7,8]. Diamine oxidase (DAO) is the main enzyme for degradation of ingested histamine [9,10], and DAO deficiency in terms of lower serum DAO levels or altered DAO activity result in an excess of histamine that may cause multifaceted symptoms due to the ubiquitous distribution of histamine receptors in organs and tissues [11,12]. DAO is an enzyme encoded by the amine oxidase copper-containing 1 (*AOC1*) gene (OMIM 104610) located in chromosome 7 (7q34-q36) in the human genome [13]. Single nucleotide polymorphisms (SNPs) of the *AOC1* gene have been recognized as the cause of DAO enzyme deficiency. The three most relevant variants in Caucasian individuals leading to a reduction in DAO enzyme activity are c.47C > T (rs10156191), c.995C > T (rs1049742), and c.1990C > G (rs1049793) [14,15], with frequencies of 24.5 % (95 % confidence interval [CI] 20.16–30.58 %), 6.3 % (95 % CI 3.42–9.26 %), and 30.6 % (95 % CI 25.1–36.1 %), respectively [14]. Moreover, another SNP in the promoter region of the *AOC1* gene has been identified, c.691G > T (rs2052129) with a frequency of 41.7 %, which has been associated with a decrease in DAO transcriptional activity [16].

The prevalence of DAO enzyme deficiency of genetic origin in the general population has not been established, but in a recent prospective population-based sample of 200 healthy newborns, alterations in at least one variant of the *AOC1* gene were present in 132, with a prevalence of 66 % (95 % CI 59–73 %) [17]. High prevalences of DAO enzyme deficiency based on alterations in the most common SNPs of the *AOC1* gene have been reported in cohort studies of specific diseases, including a prevalence of 88 % in patients with at least moderate low urinary tract symptoms (LUTS) [18], 74.5 % in women with fibromyalgia [19], and 78.5 % in pediatric patients with attention deficit hyperactivity disorder (ADHD) [20]. Sleep disturbances with low sleep quality and sleep efficiency are common prominent symptoms in all these three disorders [[21], [22], [23]].

Interestingly, a recent study carried out in a sample of 167 adult patients with symptoms of insomnia who underwent genotyping analysis of the four most relevant SNP variants of the *AOC1* gene, DAO enzyme deficiency was present in 138 patients, with a prevalence rate of 82.6 % (95 % CI 76–88.1 %) [24]. The most common clinical complaints were difficulties in staying asleep in 88 % of patients and trouble in falling asleep in 60.5 %. Also, more than half of the patients suffered from insomnia-related symptoms every day and 99.4 % reported daytime consequences of insomnia, with fatigue, mood changes, and impaired concentration [24]. The findings of this real-world study added evidence of a potential link between insomnia symptoms and DAO enzyme deficiency of genetic origin. Therefore, it was hypothesized that DAO dietary supplementation may be a useful strategy for improving sleep problems in patients with insomnia symptoms who were carriers of SNPs of the *AOC1* gene.

The objective of the study was to assess the effect of DAO enzyme dietary supplementation on chronic insomnia symptoms measured with validated sleep questionnaires in patients with impaired sleep who also presented at least one of the four SNP variants, --c.47C > T (rs10156191), c.995C > T (rs1049742), c.1990C > G (rs1049793), and c.691G > T (rs2052129)-- of the *AOC1* gene associated with a reduction of the enzymatic activity of DAO or decreased transcription of this enzyme.

## 2 Methods

### 2.1 Study design and participants

This was a prospective, randomized, double-blind, placebo-controlled and single-center study conducted at AdSalutem Sleep Institute in Barcelona, Spain. AdSalutem is a specialized and reference center in sleep disorders that provides a wide range of diagnostic and treatment modalities for adult and pediatric populations with sleep disturbances. Between March and December 2024, all consecutive eligible patients who signed the written informed consent were included in the study. Inclusion criteria were as follows: subjects of both genders; 18 years of age or older; presenting with insomnia symptoms defined according to the ICSD-3-TR diagnostic criteria [1]; evidence by genotyping analysis of being carriers, either heterozygous or homozygous, of at least one of the four SNPs of the *AOC1* gene, --c.47C > T (rs10156191), c.995C > T (rs1049742), c.1990C > G (rs1049793), and c.691G > T (rs2052129)-- associated with a reduction in the enzymatic activity of DAO or a decreased DAO transcriptional activity; and ability to complete the study questionnaires and procedures. Exclusion criteria were the presence of insomnia-related symptoms caused by other diseases, such as narcolepsy, parasomnia, circadian rhythm disturbances, obstructive sleep apnea, restless legs syndrome, etc.; history of low-histamine diet or use of DAO dietary supplementation within the last month prior to the study; and allergies to some components of the study product.

The study protocol was approved by the Clinical Research Ethics Committee (CEIC) of Grupo Hospitalario QuirónSalud-Catalunya (code DAO-SLEEP-2023, approval date December 19, 2023), Sant Cugat del Vallés, Barcelona (Spain) and was registered in the ClinicalTrials.gov (NCT07027943). All participants provided written informed consent.

### 2.2 Genotyping

We measured the four most relevant SNP variants of the *AOC1* gene through the DAO-Test® Genotyping Assay kit (DR Healthcare, Barcelona, Spain). Saliva samples from the oral mucosa were collected by rubbing the inner side of each cheek using a sterile cotton swab. The genotyping was performed with a Multiplex (Single-Nucleotide Primer Extension) SNuPE (Thermo Fisher Scientific, Applied Biosystems, Waltham, MA, USA) followed by capillary electrophoresis in an ABI 3500 Genetic Analyzer (Thermo Fisher Scientific).

### 2.3 Study procedures

Participants were randomized (1:1:1:1) to one of the four treatment arms by an independent statistician in a block size of 4 using the PROC PLAN procedure from SAS® software version 9.4 (SAS Institute, Cary, NC, USA). The study arms included dietary supplementation with the active DAO enzyme product, taken three times a day or once a day, and identically appearing placebo tablets following the same administration schedule.

The active product was DAOfood® (DR Healthcare, Barcelona, Spain), a DAO enzyme of porcine origin, authorized and regulated by the European Commission according to the Novel Food legislation of the year 2017 as a food supplement for the dietary management of DAO enzyme deficiency. DAO tablets are gastro-resistant, each tablet contains 4.2 mg of porcine kidney protein extract with an enzymatic activity of around 0.61 mU/g DAO (30,000 histamine degrading units [HDU]/g DAO). The recommended daily dose is 12.6 mg, administered as three tablets taken 20 min before each main meal (breakfast, lunch, and dinner). A second dosing regimen, consisting of the same daily dose (12.6 mg) given as three tablets taken together 20 min before dinner, was also evaluated to determine whether the timing of administration had any influence on insomnia-related symptoms. Placebo tablets contained microcrystalline cellulose and hydroxypropyl cellulose and had the same organoleptic properties as the active product.

The duration of the study was 28 days, and included a baseline visit on day 0 followed by three visits on days 7, 14, 21, and a final visit on day 28 at the end of the study. The baseline visit and the visit at the end of the study were conducted in person, whereas visits on days 7, 14, and 21 were performed via telephone. At the baseline visit, eligibility criteria were checked, the informed consent was signed, study data were collected, participants completed the Insomnia Severity Index (ISI) scale and the Pittsburg Sleep Quality Index (PSQI) questionnaire, and 100 tablets of the assigned study product were provided (90 tablets as per the protocol requirements and an additional 10 % to cover possible incidents). Also, a peripheral venous blood sample was obtained for measurement of serum levels of DAO enzyme activity. Telephone visits on days 7, 14, and 21 included questioning adverse effects (AEs), collection of study variables, and completion of the ISI scale. At the final visit, participants completed the ISI and PSQI questionnaires, compliance with the study product was checked, AEs were registered, and a second blood sample was drawn for the assessment of serum levels of DAO enzyme activity. Compliance was defined as consumption of at least 80 % of tablets, so that only 16 tablets (plus the 10 extra tablets) could be left in the blisters.

### 2.4 Study variables

For all participants the following data were collected: demographics (age, sex); body mass index (BMI); comorbidities including fibromyalgia, migraine, ADHD, depression disorder, anxiety disorder, major digestive complaints, allergy, and other; insomnia-related symptoms including difficulty falling asleep, difficulty staying asleep, walking up too early and difficulty being unable to fall back asleep; frequency of insomnia symptoms (<3 times/week, ≥3 times/week, all nights); daytime symptoms due to insomnia; use of sleep medication during the 7 days prior to the study and throughout the study period; genotype variants of the *AOC1* gene and homozygous and heterozygous state; ISI scores; PQSI scores; serum DAO enzyme activity; compliance with treatment; and AEs. The dosing group to which participants were assigned was also recorded.

The ISI is a 7-item self-report questionnaire, with a usual recall period of the last month, designed to assess the severity of both nighttime and daytime components of insomnia on a 5-point Likert scale (from 0: no problem to 4: very severe problem). The total score ranges from 0 to 28, with 0–7 interpreted as absence of insomnia, 8–14 subthreshold insomnia, 15–21 clinical insomnia (moderate severity), and 22–28 clinical insomnia (severe). A Spanish validated version was used [25]. The PSQI is a self-rated 19-item questionnaire that assesses sleep quality over an interval of 1 month. These items are combined into seven components (subjective sleep quality, sleep latency, sleep duration, habitual sleep efficiency, sleep disturbances, use of sleeping medication, and daytime dysfunction), which are scored from 0 (no difficulty) to 3 (severe difficulty). The overall score ranges between 0 and 21, with higher scores indicating worse sleep quality. A Spanish validated version of the PSQI was used [26]. Serum DAO enzyme activity was analyzed using a radioextraction assay (REA-3H, Laboratorio Echevarne, Barcelona, Spain), with >12.54 U/mL (>80 HDU/mL) as the reference value.

### 2.5 Endpoints

The primary endpoint was the improvement of insomnia symptoms of at least 1 point in the ISI scale in the active supplementation group versus placebo, given that this incremental benefit over the patients’ usual management options was deemed clinically meaningful. Secondary endpoints included the comparison between the study groups regarding: a) improvement of insomnia symptoms (of at least 1 point and overall) in the ISI scale stratified by use of dosing regimen and use of sleep medication during the study; b) improvement of insomnia symptoms (of at least 1 point and overall) in the PQSI questionnaire stratified by dosing regimen and use of sleep medication; c) improvement of the different dimensions of the PQSI questionnaire; d) changes of serum DAO activity levels; e) compliance with the study product, safety, and tolerability.

### 2.6 Statistical analysis

This study was planned as a pilot study because there were no reliable data on DAO supplementation in subjects with insomnia that would allow to calculate a powered sample size. A total of 100 patients were planned, 50 in each group. This sample size is similar to those found for studies done with the same purpose: the management of insomnia with dietary supplements, such as melatonin, probiotics or other dietary ingredients [[27], [28], [29]]. In these studies, significant changes are achieved in the same endpoints of study that our study considered (ISI and PSQI), and the duration of the intervention was in the range of 4–8 weeks [[27], [28], [29]]. The recruitment capacity of the study centre and a drop-out rate of 10 % was also considered in the calculation of the sample size. Data were analyzed in the per-protocol (PP) population, that is, subjects who met the inclusion criteria and completed at least one clinical evaluation after starting dietary supplementation with the assigned product. An analysis in the intent-to-treat (ITT) population (i.e., subjects randomized to the study arms independently of whether treatment was completed or not) was also performed. Safety data were evaluated in all randomized patients who received at least one dose of the assigned treatment.

Categorical variables are expressed as frequencies and percentages, and continuous variables as mean and standard deviation (SD). Categorical variables were compared with the Fisher's exact test or the McNemar's test, and continuous variables with the Student's *t-*test or the Wilcoxon rank-sum test according to conditions of application. Changes in sleep efficiency were calculated as the difference between post-supplementation and baseline values. Within-group differences were analyzed using paired Student's t-tests, and between-group comparisons were assessed using independent Student's t-tests. Other subgroup analyses by dosing regimens and use of sleeping medications, including melatonin and centrally acting drugs causing sedation and/or drowsiness, were pre-specified in the study protocol. Statistical significance was set at *p* < 0.05. The SAS® statistical package version 9.4 (SAS Institute Inc., Cary, NC, USA9 or the R software version 4.4.2 (RStudio IN., Boston, MA, USA) were used for statistical analysis.

## 3 Results

### 3.1 Study participants

**Fig. 1:**
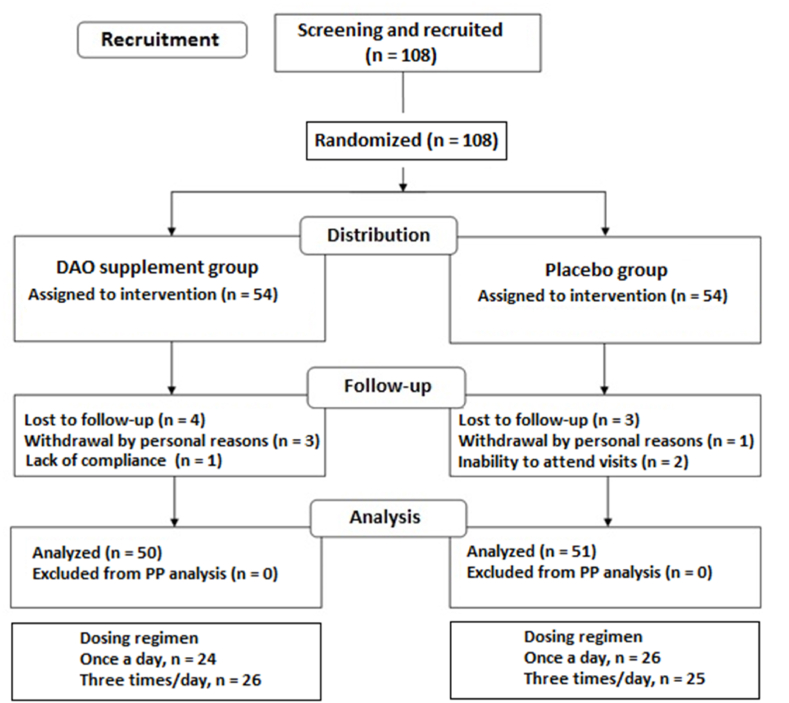
Flow chart of the study population (intent to treat [ITT] and safety populations, n = 108, DAO group n = 54, placebo group n = 54; per-protocol population [PP], n = 101, DAO group n = 50, placebo group n = 51).

Of a total of 108 eligible patients, 54 were randomized to the DAO supplementation group and 54 to the placebo group, but 7 patients (4 in the DAO supplementation group and 3 in the placebo group) were excluded from the PP population. Therefore, the study population included 101 patients, 50 in the DAO group, and 51 in the placebo group. The distribution of patients according to dosing regimens (once a day vs. three times daily) was similar (Fig. 1).

Most patients were women (74.3 %) with a mean (SD) age of 48.3 (13.0) years and BMI of 27.6 (4.0) kg/m^2^. The distribution of baseline data in the two study groups is shown in Table 1. Digestive complaints, anxiety disorder, and allergies were the most frequent comorbidities. Daytime repercussions due to insomnia and difficulty to maintain sleep were the most common symptoms. Also, 63.4 % of patients reported insomnia problems every night. Mutations in the c.47C > T (rs10156191) and c.691G > T (rs2052129) variants were documented in 82.2 % and 79.2 % of patients, respectively. A high percentage of patients reported using sleep medications in the 7 days before entering the study, including centrally acting drugs that may cause sedation and/or drowsiness (51.5 %) and melatonin (35.6 %). The mean serum DAO enzyme level was 19.4 (14.3) U/mL and only 1 % of patients showed levels <3.74 U/mL, which were suggestive of high incidence of histamine intolerance. The mean (SD) ISI score was 15.8 (4.6), with 48.5 % of patients presenting clinical insomnia of moderate severity and 12.9 % severe clinical insomnia. Results of the PSQI questionnaire also revealed poor sleep quality with a mean overall score of 12.9 (3.0) and high mean scores in the different components of the instrument.

**Table 1:** Baseline data of the study population.

Variables	All patients (n = 101)	Placebo group (n = 51)	DAO supplement (n = 50)
Female patients, n (%)	75 (74.3)	42 (82.3)	33 (66.0)
Age, years, mean (SD)	48.3 (13.0)	47.9 (13.1)	48.7 (13.0)
Body mass index, BMI, kg/m^2^, mean (SD)	23.6 (4.0)	23.2 (3.4)	23.9 (4.5)
Comorbidities, n (%)			
Digestive complaints	52 (51.5)	29 (56.9)	23 (46.0)
Anxiety disorder	51 (50.5)	29 56.9)	22 (44.0)
Allergy	42 (41.6)	20 (39.2)	22 (44.0)
Depressive disorder	27 (26.7)	15 (29.4)	12 (24.0)
Migraine	21 (20.8)	10 (19.6)	11 (22.0)
Fibromyalgia	21/20.8)	10 (19.6)	11 (22.0)
Attention deficit hyperactivity disorder (ADHD)	7 (6.3)	5 (9.8)	2 (4.0)
Insomnia-related symptoms, n (%)			
Difficulty falling asleep	61 (60.4)	30 (58.8)	31 (62.0)
Difficulty to maintain sleep	88 (87.3)	43 (84.3)	45 (90.0)
Early morning awakening and inability to go back to sleep	79 (78.2)	37 (72.5)	42 (84.0)
Daytime repercussion due to insomnia	95 (94.1)	49 (96.1)	46 (92.0)
Frequency of symptoms			
<3 times/week	4 (4.0)	2 (3.9)	2 (4.0)
≥3 times/week	33 (32.7)	16 (31.4)	17 (34.0)
All nights	64 (63.4)	33 (64.7)	31 (62.0)
Genotype variants, n (%)			
c.47C > T (rs10156191) CC	20 (19.8)	14 (27.5)	6 (12.0)
CT/TT	81 (80.2)	37 (72.5)	44 (88.0)
c.995C > T (rs1049742) CC	47 (46.5)	23 (45.1)	24 (48.0)
CT/TT	54 (53.5)	28 (54.9)	26 (52.0)
c.1990C > G (rs1049793) CC	59 (58.4)	32 (62.7)	27 (54.0)
CG/GG	42 (41.6)	19 (37.2)	23 (46.0)
c.691G > T (rs2052129) GG	21 (20.8)	12 (23.5)	9 (18.0)
GT/TT	80 (79.2)	39 (76.4)	41 (82.0)
Use of sleeping medications in the previous 7 days, n (%)	69 (68.3)	34 (66.7)	35 (70)
Centrally acting drugs causing sedation and/or drowsiness	52 (51.5)	25 (49.0)	27 (54)
Melatonin	36 (35.6)	15 (29.4)	21 (42)
Phytotherapy	7 (6.9)	6 (11.8)	1 (2)
Serum DAO enzyme, U/mL, mean (SD)	19.4 (14.3)	19.2 (14.3)	19.6 (14.5)
>12.54 U/mL, n (%)	64 (63.4)	29 (56.9)	35 (70)
3.74–12.54 U/mL, n (%)	36 (35.6)	21 (41.2)	15 (30)
<3.74 U/mL, n (%)	1 (1.0)	1. (1.0)	0
Insomnia Severity Index (ISI) overall score, mean (SD)	15.77 (4.62)	16.41 (4.37)	15.12 (4.82)
No clinically significant insomnia (scores 0–7), n (%)	2 (1.9)	1 (1.0)	1 (2)
Subthreshold insomnia (scores 8–14), n (%)	37 (36.6)	16 (31.4)	21 (42)
Clinical insomnia (moderate severity) (scores 15–21), n (%)	49 (48.5)	27 (52.9)	22 (44)
Clinical insomnia (severe) (scores 22–28), n (%)	13 (12.9)	7 (13.7)	6 (12)
Pittsburgh Sleep Quality Index (PSQI), overall score, mean (SD)	12.93 (2.84)	12.94 (2.67)	12.92 (3.02)
Subjective sleep quality, mean (SD)	2.10 (0.59)	2.14 (0.57)	2.06 (0.62)
Sleep latency, mean (SD)	1.65 (1.09)	1.53 (1.12)	1.78 (1.06)
Sleep duration, mean (SD)	2.0 (0.57)	2.0 (0.66)	2.0 (0.45)
Sleep efficiency, mean (SD)	1.90 (1.03)	1.92 (1.13)	1.88 (0.94)
Sleep disturbances, mean (SD)	1.36 (0.50)	1.35 (0.52)	1.36 (0.48)
Use of sleeping medication, mean (SD)	1.94 (1.33)	1.84 (1.36)	2.04 (1.29)
Daytime dysfunction, mean (SD)	1.95 (1.02)	2.10 (0.98)	1.80 (1.05)

DAO: diamine oxidase; SD: standard deviation.

### 3.2 Improvement of insomnia-related symptoms

At the end of the study, there was not a statistically significant difference in between-groups comparison regarding the percentage of patients who showed an improvement of at least 1 point in the ISI questionnaire. As shown in Table 2, there were statistically significant differences in the within-group comparisons of mean decreases of ISI scores over the study period, but between-group comparisons were not significant. The mean decrease on day 28 as compared with baseline was −3.45 for the overall study population, −4.39 (5.64) for the placebo group and −2.48 (4.49) for the DAO supplementation group (*p* = 0.0622). The percentage of patients who at the end of the study showed ISI scores 22–28 corresponding to clinical severe insomnia was higher in the placebo group than in the DAO supplementation group (5.9 % vs. 2 %) but differences were not significant (*p* = 0.7971). Moreover, the number of patients who reported no clinically significant insomnia at baseline increased from 2 (2.0 %) to 16 (15.8 %) at the end of the study, with similar increases in both study groups.

**Table 2:** Changes of Insomnia Severity Index (ISI) scores during the study period as compared with baseline.

Time periods	All patients (n = 101)			Placebo group (n = 51)			DAO supplementation (n = 50)			Between-group *p* value
	Mean (SD)	Mean (SD) change vs. baseline	Within-group *p* value	Mean (SD)	Mean (SD) change vs. baseline	Within-group *p* value	Mean (SD)	Mean (SD) change vs. baseline	Within-group *p* value	
Baseline	15.77 (4.62)			16.41 (4.37)			15.12 (4.82)			
Day 7	13.01 (4.61)	−2.76 (4.25)	<0.0001	12.94 (4.54)	−3.47 (4.62)	<0.0001	13.08 (4.73)	−2.04 (3.75)	0.003	0.090
Day 14	12.82 (4.85)	−2.95 (4.52)	<0.0001	12.94 (5.05)	−3.47 (4.44)	<0.0001	12.70 (4.68)	−2.42 (4.58)	0.0005	0.244
Day 21	12.88 (5.39)	−2.89 (5.08)	<0.0001	13.16 (5.49)	−3.25 (5.48)	0.0001	12.60 (5.33)	−2.52 (4.67)	0.0004	0.469
Day 28	12.33 (5.09)	−3.45 (5.17)	<0.0001	12.02 (5.29)	−4.39 (5.64)	<0.0001	12.64 (4.91)	−2.48 (4.49)	0.0003	0.062

SD: standard deviation; DAO: diamine oxidase.

**Fig. 2:**
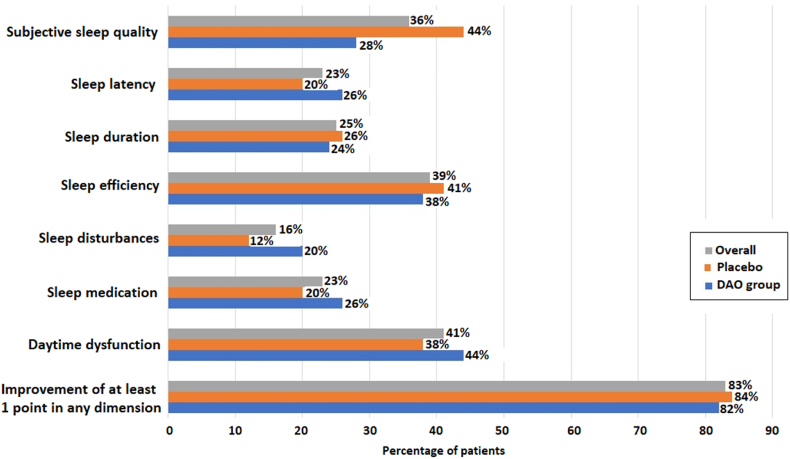
Percentages of patients with improvement of at least 1 point at the end of the study as compared with baseline in the different dimensions of the Pittsburgh Sleep Quality Index (PSQI) questionnaire (DAO: diamine oxidase).

In relation to changes of insomnia-related symptoms evaluated with the PSQI questionnaire at the end of the study (Table 3), there were statistically significant decreases in the overall mean scores in both study groups, but the mean within-group improvement was higher in the DAO supplementation group as compared with placebo (−1.62 [3.45] vs. −1.47 [3.21], *p* = 0.8225). Also, in other components of the questionnaire, including sleep latency, sleep duration, sleep efficiency, use of sleep medication, and daytime dysfunction, mean changes on day 28 as compared with baseline were higher in the DAO supplementation group than in the placebo group, but between-group differences were not statistically significant (Table 3). Additionally, sleep efficiency, as assessed by the PSQI, improved significantly from baseline after 28 days of DAO supplementation (*p* = 0.0165), but not after placebo (*p* = 0.1423). The mean (SD) improvement in sleep efficiency was 0.052 (0.15) in the DAO group and 0.034 (0.16) in the placebo group. However, the difference between treatment groups was not statistically significant. Fig. 2 shows the percentage of patients who showed an improvement of at least 1 point in the PSQI questionnaire on day 28 as compared with baseline. In the DAO supplementation group, there were higher percentages of patients with improvements in sleep latency, sleep disturbances, use of sleep medication, and daytime dysfunction, whereas subjective sleep quality, sleep efficiency, and sleep duration were higher in the placebo group.

**Table 3:** Changes of the Pittsburgh Sleep Quality Index (PSQI) scores during the study period as compared with baseline.

Items	Baseline mean (SD)	Day 28 mean (SD)	Change mean (SD)	Within-group *p* value	Between-group *p* value
Overall (n = 99)	12.93 (2.84)	11.38 (3.56)	−1.55 (3.32)	<0.0001	0.822
Placebo (n = 49)	12.94 (2.67)	11.47 (3.67)	−1.47 (3.21)	0.0024	
DAO group (n = 50)	12.92 (3.02)	11.30 (3.49)	−1.62 (3.45)	0.0017	
Subjective sleep quality					
All patients (n = 100)	2.09 (0.59	1.83 (0.64)	−0.26 (0.75)	0.0002	0.220
Placebo (n = 50)	2.12 (0.56)	1.76 (0.66)	−0.36 (0.83)	0.004	
DAO group (n = 50)	2.06 (0.62)	1.90 (0.61)	−0.16 (0.65)	0.092	
Sleep latency					
All patients (n = 100)	1.64 (1.09)	1.55 (1.05)	−0.09 (0.82)	0.295	0.441
Placebo (n = 50)	1.50 (1.11)	1.46 (0.99)	−0.04 (0.83)	0.840	
DAO group (n = 50)	1.78 (1.06)	1.64 (1.10)	−0.14 (0.81)	0.227	
Sleep duration					
All patients (n = 100)	2.0 (0.57	1.85 (0.67)	−0.15 (0.70)	0.034	0.818
Placebo (n = 50)	2.0 (0.67)	1.88 (0.66)	−0.12 (0.69)	0.228	
DAO group (n = 50)	2.0 (0.45)	1.82 (0.69)	−0.18 (0.72)	0.083	
Sleep efficiency, %					
All patients (n = 99)	1.91 (1.02)	1.51 (1.26)	−0.40 (1.22)	0.001	0.854
Placebo (n = 49)	1.94 (1.11)	1.57 (1.27)	−0.37 (1.20)	0.036	
DAO group (n = 50)	1.88 (0.94)	1.44 (1.26)	−0.44 (1.25)	0.015	
Sleep disturbances					
All patients (n = 100)	1.36 (0.50)	1.26 (0.48)	−0.10 (0.46)	0.034	0.763
Placebo (n = 50)	1.36 (0.53)	1.24 (0.48)	−0.12 (0.33)	0.019	
DAO group (n = 50)	1.36 (0.48)	1.28 (0.50)	−0.08 (0.57)	0.331	
Use of sleep medication					
All patients (n = 100)	1.93 (1.33)	1.71 (1.37)	−0.22 (1.08)	0.049	0.433
Placebo (n = 50)	1.82 (1.37)	1.68 (1.36)	−0.14 (1.01)	0.369	
DAO group (n = 50)	2.04 (1.29)	1.74 (1.38)	−0.30 (1.15)	0.075	
Daytime dysfunction					
All patients (n = 100)	1.94 (1.02)	1.64 (0.98)	−0.30 (1.11)	0.011	0.8405
Placebo (n = 50)	2.08 (0.99)	1.80 (0.99)	−0.28 (1.07)	0.087	
DAO group (n = 50)	1.80 (1.05)	1.48 (0.95)	−0.32 (1.17)	0.058	

SD: standard deviation; DAO: diamine oxidase.

### 3.3 Dosing regimens

Improvements of insomnia-related symptoms according to the dosing regimens did not show significant differences in the ISI questionnaire, although the percentage of patients who improved at least 1 point at the different study visits as compared with baseline and throughout the study period appeared to be more favorable to the once-daily regimen (Table 4). In the DAO supplementation group, 91.7 % of patients showed an improvement of at least 1 point throughout the study period in the once-a-day regimen as compared to 80.8 % in the three-times-a-day regimen. Results obtained in the PSQI questionnaire were similar, with higher percentages of patients with improvements of at least 1 point in the once-daily regimen, particularly in the components of sleep duration, sleep efficiency, and sleep disturbances.

**Table 4:** Percentage of patients with improvement of at least 1 point in the study questionnaires as compared with baseline according to dosing regimens.

Variables		Once a day		Three times daily	
		Placebo group (n = 26)	DAO group (n = 24)	Placebo group (n = 25)	DAO group (n = 26)
Insomnia Severity Index (ISI)	Study visits vs. baseline				
	Day 7	76.9 %	75 %	72 %	50 %
	Day 14	76.9 %	75 %	80 %	61.5 %
	Day 21	69.2 %	54.1 %	72 %	69.2 %
	Day 28	73.1 %	70.8 %	72 %	61.5 %
	Throughout the study	96.1 %	91.7 %	92 %	80.8 %
Pittsburgh Sleep Quality Index (PSQI)	Day 28 vs. baseline				
	Subjective sleep quality	46.1 %	25 %	41.7 %	30.8 %
	Sleep latency	23.1 %	25 %	16.7 %	26.9 %
	Sleep duration	26.9 %	29.2 %	25 %	19.2 %
	Sleep efficiency	32 %	54.2 %	50 %	23.1 %
	Sleep disturbances	15.4 %	25 %	8.3 %	15.4 %
	Use of sleep medication	23.1 %	25 %	16.7 %	26.9 %
	Daytime dysfunction	34.6 %	33.3 %	41.7 %	53.8 %
	Throughout the study	84.6 %	70.8 %	83.3 %	92.3 %

DAO: diamine oxidase.

Moreover, in the once-daily regimen in the DAO supplementation group, the mean (SD) score of sleep efficiency decreased from 1.83 (0.92) at baseline to 1.12 (1.33) at day 28 with a mean change of −0.71 (1.43) (*p* = 0.023), but in the placebo group decreases were not significant, from 2.0 (1.12) to 1.68 (1.25) with a mean decrease of −0.32 (1.11) (*p* = 0.197). In relation to the PSQI component of use of sleep medication, within-group differences were statistically significant for the DAO group only, with a mean decrease from 2.12 (1.33) to 1.58 (1.44) and a mean change of −0.54 (1.14) (*p* = 0.043) as compared with decreases of 1.81 (1.36) to 1.58 (1.42) and a mean change of −0.23 (1.18) (*p* = 0.333) in the placebo group.

### 3.4 Sleeping medication

During the study 28 patients did not use sleeping medication, 10 used melatonin only, and 25 used melatonin and centrally acting drugs that may cause sedation and/or drowsiness. In the 28 patients who did not use any pharmacological sleep medication (13 in the placebo group and 15 in the DAO supplementation group) statistically significant changes in the ISI scores over the study period were not found in any of the study groups. In relation to the PSQI questionnaire, there was a statistically significant reduction in the overall score in the DAO supplementation group only, from a mean (SD) value of 10.87 (3.02) at baseline to 8.53 (2.39) at day 28, with a mean decrease of −2.33 (3.90) (*p* = 0.036), whereas differences in the placebo group were not significant.

In the 10 patients who used melatonin only (4 in the placebo group and 6 in the DAO supplementation group), results of the ISI questionnaire showed a significant improvement in insomnia-related symptoms at 7 days of treatment in both study groups (mean change −5.75 [2.06] in the placebo group, *p* = 0.011, vs. −4.17 [2.23] in the DAO supplementation group, *p* = 0.005), being superior in the DAO group. This significant improvement was maintained until day 14 in the DAO supplementation group only and remained until the end of the study (Table 5). In the PSQI questionnaire, statistically significant differences were observed in the DAO group only regarding improvements in the overall score and in the component of sleep efficiency (Table 5). In relation to improvement of at least 1 point in the PSQI questionnaire, 33.3 % and 50 % of patients in the DAO group showed improvement in sleep latency and sleep duration, respectively, whereas none of the patients in the placebo group showed improvements in these components. Sleep efficiency improved in 66.7 % of patients in the DAO group as compared to 50 % in the placebo group.

**Table 5:** Changes of insomnia-related symptoms in patients using sleeping medications.

Patients taking melatonin only (n = 10)							
Time periods		Placebo (n = 4)			DAO supplementation (n = 6)		
		Mean (SD) score	Mean (SD) change vs. baseline	*P* value	Mean (SD) score	Mean (SD) change vs. baseline	*P* value
Insomnia Severity Index (ISI)	Study visits						
	Baseline	19.75 (3.30)			14.0 (3.29)		
	Day 7	14.0 (2.31)	−5.75 (2.06)	0.011	9.83 (2.99)	−4.17 (2–23)	0.006
	Day 14	13.50 (5.92)	−6.25 (4.03)	0.053	8.50 (3.51)	−5.50 (4.32)	0.026
	Day 21	14.50 (4.65)	−5.25 (3.69)	0.065	9.50 (3.33)	−4.50 (4.37)	0.053
	Day 28	13.0 (3.46)	−6.75 (6.40)	0.125	10.67 (3.14)	−3.33 (3.98)	0.095
Pittsburgh Sleep Quality Index (PSQI)	Components						
	Overall score						
	Baseline	13.50 (2.08)			12.67 (2.42)		
	Day 28	13.0 (2.16)	−0.50 (2.08)	0.663	10.17 (2.32)	−2.50 (1.97)	0.026
	Sleep efficiency						
	Baseline	2.25 (0.96)			1.67 (1.03)		
	Day 28	1.75 (1.26)	−0.50 (0.58)	0.345	0.67 (1.21)	−1.0 (0.89)	0.049

Patients taking melatonin and centrally acting drugs causing sedation and/or drowsiness (n = 25)							
Time periods		Placebo (n = 12)			DAO supplementation (n = 13)		
		Mean (SD) score	Mean (SD) change vs. baseline	*P* value	Mean (SD) score	Mean (SD) change vs. baseline	*p* value
Insomnia Severity Index (ISI)	Study visits						
	Baseline	15.50 (5.20)			16.23 (4.30)		
	Day 7	12.58 (3.63)	−2.92 (5.28)	0.082	15.69 (3.99)	−0.54 (4.22)	0.653
	Day 14	14.50 (5.66)	−1.0 (3.16)	0.296	14.92 (3.35)	−1.31 (4.48)	0.313
	Day 21	14.92 (6.36)	−0.58 (4.10)	0.631	13.23 (4.51)	−3.0 (4.18)	0.023
	Day 28	14.75 (5.61)	−0.75 (5.07)	0.618	14.54 (4.61)	−1.69 (4.29)	0.180
Pittsburgh Sleep Quality Index (PSQI)	Components						
	Overall score						
	Baseline	14.33 (3.17)			14.31 (2.53)		
	Day 28	14.42 (3.0)	0.08 (2.75)	0.918	13.54 (3.04)	−0.77 (2.77)	0.714
	Sleep efficiency						
	Baseline	2.08 (1.08)			2.15 (0.90)		
	Day 28	2.08 (1.16)	0.0 (1.28)	1.0	2.15 (1.14)	0.0 (0.82)	1.0

There were 25 patients (13 in the placebo group, 12 in the DAO supplementation group) who reported the use of melatonin associated with centrally acting drugs causing sedation and/or drowsiness, mostly benzodiazepines and non-benzodiazepine hypnotics. In these patients, significant improvements in the ISI questionnaire were observed on day 21 in the DAO group only (Table 5). Changes in the scores of the PSQI questionnaire were not significant in any of the study groups, although the percentage of patients who improved at least 1 point over the study period was 92.3 % in the DAO supplementation group and 88.3 % in the placebo group. Also, there were higher percentages of improvement in patients in the DAO group as compared to placebo in the components of sleep disturbances (38.5 % vs. 8.3 %) and daytime dysfunction (46.1 % vs. 16.7 %), although differences were not statistically significant.

Results obtained in all study variables in the ITT dataset were similar than those found in the PP population (data not shown).

### 3.5 Compliance, tolerability and safety

All participants consumed at least 80 % of the assigned dietary supplement. AEs related to the study product were not registered and serios AEs did not occur. Four participants randomized to the DAO supplementation group presented 5 AEs, and 11 assigned to the placebo group reported AEs. All AEs were of mild or moderate intensity, with headache (5.5 %) and gastrointestinal complaints (4.6 %) as the most frequent. Other AEs, such as fatigue, edema, pruritus, blurred vision, flu-like symptoms, and paresthesia were reported in one case each.

## 4 Discussion

In this randomized, double-blind, placebo-controlled trial conducted in patients with chronic insomnia and SNP variants of the *AOC1* gene, consumption of a food supplement containing a porcine-derived DAO enzyme for 28 consecutive days was associated with significant improvements in the subjective perception of better sleep quality as compared with placebo. These findings are clinically relevant to establish a link between insomnia symptoms and genetic alterations of the *AOC1* gene, potentially manageable by a simple dietary strategy based on DAO enzyme supplementation.

Changes in insomnia-related symptoms were evaluated using the ISI and PSQI questionnaires, which are validated and widely recognized instruments to assess signs and symptoms of insomnia as well as changes across various domains of sleep quality.

In relation to the primary endpoint of the study (i.e., improvement of insomnia symptoms of at least 1 point in the ISI scale in the DAO supplementation group versus placebo), improvements at the different time points of the study as compared with baseline were statistically significant within each study group, but statistical significance for between-group comparisons was not found. On the other hand, although a higher percentage of patients in the placebo group than in the DAO supplementation group reported clinical severe insomnia at the end of the study, significant differences were not observed. However, despite these similarities in improvements of insomnia symptoms as measured by the ISI, when the two dosing regimens were compared, the once-daily schedule appeared to be superior to three times daily dosing regimen in higher percentages of patients achieving at least 1 point of improvement, particularly on days 7, 14, and 28, as well as throughout the study in the DAO supplementation group.

Results obtained using the PSQI questionnaire showed significant differences in the within-group comparisons for the overall score and the seven subscales, with generally higher within-group differences in the DAO supplementation group as compared with the placebo group, although between-group significant differences were not observed. Other interesting findings included a higher mean score reductions of sleep latency, sleep duration, sleep efficiency, use of sleep medication, and daytime dysfunction on day 28 as compared with baseline in the DAO supplementation group. Moreover, sleep duration, sleep efficiency, and sleep disturbances were the components with higher percentages of patients assigned to the DAO supplementation group who improved at least 1 point in the PSQI questionnaire. Interesting, sleep efficiency and use of sleep medication showed statistically significant within-group differences in the DAO group only for the dosing regimen of once-daily, whereas within-group differences in the placebo group did not reach statistical significance.

All these findings may indicate that the use of a dietary DAO supplement, administered once a day at night (20 min before dinner), was associated with potential higher benefits especially for improving sleep efficiency. Sleep efficiency is a crucial indicator of sleep quality and there is strong evidence of the implications of poor sleep quality and/or sleep duration on a range of adverse health consequences and overall well-being, including psychosocial, occupational and economic repercussions, as well increasing the risk of accidents and the development of depression, anxiety, and substance-related problems [30,31]. Promoting education on healthy sleep in both pediatric and adult populations remain a significant unmet need.

This study also examined the potential implications of the concurrent use of sleeping medications on the effect of dietary DAO supplementation on insomnia-related symptoms, although the limited samples sizes in the different subgroups should be considered at the time of interpreting the findings of this exploratory analyses. There were 28 patients (27.7 %) who did not use any sleeping medication over the course of the study. In these patients, any potential confounding influence of such agents on the potential benefits of the study products was absent. The use of sleeping aids was neither an exclusion criterion nor a forbidden treatment during the study because it was intended to replicate real-world conditions and to explore a possible additive effect of DAO supplementation and sleeping medications. Despite the benefits of medications for the treatment of chronic insomnia, there is ongoing debate regarding their appropriate drug selection and duration of use [32].

In the subset of patients without sleeping medication, a statistically significant decrease in the overall score of the PSQI at the end of the study in the DAO supplementation group only was clearly shown. Additionally, in patients who consumed melatonin, there were significant improvements in the ISI questionnaire, particularly at day 14, and in the PSQI overall score and sleep efficiency subscale among patients assigned to the DAO supplementation group. The clinical relevance of this potential synergy between melatonin and dietary DAO supplementation merits further investigation in populations for whom melatonin is indicated or recommended, such as those with primary sleep disorders [33] or ADHD [34,35], among other clinical conditions. Finally, in patients using melatonin together with centrally acting drugs causing sedation and/or drowsiness, significant ISI improvement by day 21 was observed only in the DAO group. Also, the percentage of patients who improved at least 1 point in the PSQI questionnaire was higher in the DAO supplementation group. Additional benefits in the DAO group were noted in sleep disturbances and daytime dysfunction.

The present findings should be interpreted considering the limitations of the study, in particular the single-center design and the small number of patients included in some of the subgroup analysis. Also, the ISI and PSQI questionnaires are focused on assessment of the subjective symptoms of insomnia and other objective methods, such as actigraphy or polysomnography were not used. However, subjective perception of the quality of sleep is a critical factor because diagnosis and clinical severity of sleep disturbances are primarily based on the patient's reported experience, rather than in objective findings from sleep studies or other physiological measurements. Moreover, many patients with insomnia have a substantial discrepancy between the amount of sleep perceived and objectively registered, a phenomenon known as “sleep state misperception” or “subjective-objective discrepancy” [[36], [37], [38]]. It should be noted that comorbidities (e.g., anxiety and depressive disorders, allergy, migraine and fibromyalgia) were frequent in the study population, although the baseline distribution of patients with concomitant disease was similar in the DAO supplementation and the placebo groups. However, a subanalysis of patients with and without comorbidities was not performed.

## 5 Conclusions

This exploratory study shows that in patients with insomnia-related symptoms and SPNs of the *AOC1* gene, which encodes the DAO enzyme, the administration of a dietary DAO supplement for 28 days was associated with improvement of sleep symptoms. Statistically significant within-group decreases of mean scores of the ISI a PSQI questionnaire throughout the study period were observed in the two study groups, but the magnitude of the differences was higher in the DAO supplementation group. Improvements in sleep efficiency were particularly noticeable in the DAO supplementation group. The results obtained with the once a day or three times daily dosing regimens were quite similar, although they seemed to be more favorable for the once-daily schedule. The potential synergy between melatonin and DAO supplementation appears to be a useful advantage that warrants further investigation. This study shows that dietary supplementation with DAO may be a clinically relevant approach for improving insomnia symptoms in patients with genetic anomalies of the *AOC1* gene. The present preliminary findings, however, should be confirmed in future studies with larger study samples and longer supplementation periods.

## CRediT authorship contribution statement

**Júlia Ferrer-Garcia:** Writing – review & editing, Validation, Project administration, Methodology, Investigation, Data curation, Conceptualization. **Maria D. Navarro:** Writing – review & editing, Investigation. **Montserrat Abadias:** Writing – review & editing, Methodology, Data curation, Conceptualization. **Raquel López-García:** Writing – review & editing, Supervision. **Anna Sansalvador:** Writing – review & editing, Supervision. **Georgia Gris:** Writing – review & editing, Data curation. **Karol Uscamaita:** Writing – review & editing, Supervision.

## Institutional review board statement

The study was conducted in accordance with the Declaration of Helsinki, and approved by the Clinical Research Ethics Committee (CEIC) of Grupo Hospitalario QuirónSalud Catalunya (code DAO-SLEEP-2023, approval date December 19, 2023), Sant Cugat del Vallés, Barcelona, Spain.

## Funding

DR Healthcare S.L.U., provided support for the logistic aspects of the study.

## Declaration of competing interest

The authors declare that they have no known competing financial interests or personal relationships that could have appeared to influence the work reported in this paper.

## Data Availability

The study data are available from the corresponding author upon request.
